# Food Choice Behaviors of Lactating Women: Association with Body Mass Index and Fruits and Vegetables Intake in Central Amhara Region, Ethiopia—An Observational Study

**DOI:** 10.1155/2021/6654659

**Published:** 2021-02-28

**Authors:** Gesessew Kibr

**Affiliations:** Department of Food and Nutritional Sciences, Faculty of Agriculture, Shambu Campus, Wollega University, Shambu, Ethiopia

## Abstract

**Background:**

What food people choose to eat is embraced by circumstances that are essentially influenced by the underlying motives of food choices which are important to a healthful dietary change. Therefore, this study aimed to explore food choice behaviors associated with nutritional status and FVs intake in women during lactation from central Amhara.

**Methods:**

A multistage sampling technique was applied to select study participants. A study was conducted among 423 randomly selected lactating women using a face-to-face interview of the questionnaire. A community-based cross-sectional, quantitative study with descriptive and analytical components was done. Anthropometrics measurements such as weight, height, waist, and hip circumference were taken from all participants. Body mass index was calculated to determine the nutritional status of women. Data were analyzed using SPSS version 20 computer software program. Association between dependent and independent variables was identified by logistic regression analysis, and a *P*-value< 0.05 was taken as statistically significant.

**Results:**

About 21.7% of women were underweight. Approximately 37.4% of women had good consumption trends to fruits and vegetables. Healthy meal eating motivation, price-consciousness, and mood concern were identified as the most significant behaviors of food choice, influencing the nutritional status of women with AOR (95% CI) of 2.1 (1.21–3.62), 3.01 (1.32–6.9), and 0.5 (0.30–0.95), respectively. Natural content (AOR (95%): 2.37 (1.39–4.06)), mood concern (AOR (95%): 2.29 (1.15–4.56)), religion concern (AOR (95%): 2.45 (1.37–4.4)), husband encouragement (AOR (95%): 2.26 (1.04–4.89)), and availability of milk and milk products (AOR (95%): 1.6 (1.39–2.74)) and fruits and vegetables (AOR (95%): 1.66 (1.16–3.48)) in home were associated significantly with a good intake of fruit and vegetables.

**Conclusions:**

This finding provides a useful insight into the food choice motivations of a group of women with preference for natural foods, mood concern, religion concern, and husband support with good fruit and vegetable intake. This can help practitioners make recommendations for health promotion strategies. Emphasis on assisting women with healthy meal eating and avoiding psychological stress is important. Nutrition education about healthy food choice is recommended for communities.

## 1. Introduction

Malnutrition from preventable causes excessively affects the wellbeing of millions of people, especially women and children under five years old in developing countries [1]. A change in lifestyle and working conditions apart from changing the dietary pattern hugely attributes to nutrition-related noncommunicable chronic diseases (NCDs) in developing countries too [2–5]. In Ethiopia, studies reported malnutrition in women like overweight/obesity [4, 6–9], undernutrition [4, 6, 7, 10–13], micronutrient deficiencies [14], anemia and vitamin A deficiency [11], and diet-related NCDs burden [12, 15–17]. Chronic health disorders such as obesity, diabetes, and cardiovascular diseases have been increasing in Ethiopia for the last few decades [18]. Human healthy dietary intakes are dependent on consuming enough variety of foods to provide all the required nutrients to sustain life. If food systems do not provide adequate diversity of foods to meet these needs constantly, malnutrition could be prevalent among populations [19].

Improving nutrition across the life span of the population especially reproductive-age women is essential for the well-being of families and communities and the successful economic and social advancement [2–4, 12–15, 19]. According to the Ethiopian Demographic and Health Survey, 27% of Ethiopian women are too thin, with 6% being overweight or obese [12]. According to the Federal Minister of Health (FMOH), 17% of women were anemic and 6% of women were experiencing vitamin A deficiency [11]. In Ethiopia, different studies reported the prevalence of undernutrition among women [6, 7, 9, 12], which was about 38%, 27%, 25%, and 17.4%, respectively.

According to the USAID report on Ethiopia nutrition using 2012 data, 27% of women were subjected to micronutrient deficiencies [14]. Dietary behavior like intake of a diet low in fruits and vegetables and high in sodium contributes significantly to the noncommunicable disease burden in Ethiopia [17]. A cross-sectional study in Gilgel Gibe found that 9% of the population was affected by chronic diseases [16]. The Ethiopian nationwide study reported a strong association between nutritional impairment and the development of chronic diseases such as cardiovascular diseases, cancer, and diabetes [15]. The proportion of noncommunicable disease deaths associated with low fruit consumption slightly increased from 11.3% in 1990 to 11.9% in 2013 [17]. Consumption of fruits and vegetables (FVs) is the most sustainable way of controlling micronutrient deficiencies in poor-resource communities and associated with reduced risk of health conditions/noncommunicable diseases such as obesity, diabetes, cancer, and cardiovascular diseases [19, 20]. Since they are rich in vitamins, minerals, and dietary fiber and low in calories [21], the micronutrient supplies of FVs are vital for the functioning of the gastrointestinal tract as they enable the body to use other nutrients (fats and carbohydrates) required for its normal function [22].

Based on the evidence of the role of FVs in the prevention of many health problems, the Food and Agriculture Organization (FAO) and World Health Organization (WHO) have recommended that people should eat at least 400 g of FVs per person per day or five servings a day; a standard portion size is assumed to be 80 g. However, actual portions tend to be lower for vegetables and higher for fruits, at least two servings (160 g) of fruits and three servings (240 g) of vegetables with at least one serving of vegetables involving dark green and leafy or orange vegetable (nutrient-rich vegetable) or about 146 kg per capita (per person) per year FVs combined (excluding potatoes and other starchy tubers, cassava, and sugar). However, the consumption of FVs in African countries ranged from 27 kg to 114 kg per person per year, far below the WHO/FAO minimum recommended levels. Ethiopia stands out as a country with low consumption of FVs, both in urban and rural areas with an average intake of fewer than 100 grams per day [23]. In this setting, identifying drivers of food choice associated with the actual food consumption is basic to guide the nutritional status of the target population in a healthier direction [24].

Poor dietary behavior like a diet low in FVs and high in sodium contributes significantly to the noncommunicable disease burden in Ethiopia [19]. A cross-sectional study done in Gilgel Gibe found that 9% of the population is affected by chronic diseases [18]. The Ethiopian nationwide study reported a strong association between nutritional impairment and the development of chronic diseases such as cardiovascular diseases, cancer, and diabetes [16].The proportion of noncommunicable diseases and deaths associated with low fruit consumption slightly increased from 11.3% in 1990 to 11.9% in 2013 [17].

Nutrition-related health problems are among the Ethiopian government's priorities, especially towards the attainment of the sustainable development goals, to end hunger, food insecurity, and all other forms of malnutrition [10, 11, 15]. Such low achievements could be further explained by poor food choice decisions, which remain poorly understood and resulting in focusing on less healthy foods which is a silent critical issue contributing to overall malnutrition in Ethiopia [4, 14–17]. Women are the most crucial group to produce productive and effective human power so that they should remain in a healthy status [25]. Adequate nutrition by maintaining optimal food choices throughout a woman's time and healthy lifestyle behavior is a significant consideration to improve the health of both babies and women [23–26]. Lactating women are considered as a nutritionally vulnerable group and subjected to nutritional stress due to their nursing process [27]. This physiological state increases the feeling of hunger which makes them different from other women of reproductive age like pregnant women who had a unique physiological status and nonpregnant and lactating women. Lactating women have additional nutritional recommendations [28]. Because of this nature, lactating women may have different food choice behavior compared to other childbearing aged women. In the past, several studies were conducted on food consumption patterns of lactating women in Ethiopia [7, 9, 29]. Furthermore, food choice behaviors are rapidly evolving in response to recent changes from different perspectives in the communities' livelihoods and cultural norms in the face of nutrition transition and other trends. However, no studies have been conducted for a deeper understanding of the key drivers of food choice associated with food consumption patterns and nutritional status in that nutritionally vulnerable group. Therefore, this study was designed to explore various determinants of food choice associated with nutritional status and FVs consumption among women in Ethiopia.

## 2. Materials and Methods

### 2.1. Study Setting, Design, and Period

The study was conducted in Debre Berhan Town, North Shewa Zone, Amhara Regional State, Ethiopia. Debre Berhan is a zonal town and located in central Ethiopia at a distance of 130 Km away from Addis Ababa and 695 Km away from Bahir Dar which is the regional capital. Its astronomical location is 11° 06′ North Latitude and 39° 45′ East Longitude. The topography is classified as 86% flat, 10% sloppy, and 4% mountainous. The total area of the city is 21169.95 hectares of land [15].

The dominant economic activities in the town are tanneries, blanket factories, and beverage factories. Agriculture in the suburbs provides livelihood to a large section of society. In the town, there is one government university, various colleges, schools, banks, hospitals, both public and private, various clinics, and primary, secondary, and preparatory schools. Based on the population projection from the central statistics agency of Ethiopia (2015), the town has a total population of 102,500 [30]. The majority (94.12%) of the inhabitants practiced Ethiopian Orthodox Christianity [15].

The study area was selected since it is the scarcity of researched information regarding the driver of food choice in addition to resources limitation by the researcher. All participants completed a short background questionnaire. Descriptively, the cross-sectional study design was employed to investigate the characteristics of women, FVs consumption, and specific food choice behaviors from Debre Berhan town between March and June 2016. Based on the following criteria, study participants were recruited:

  Lactating women aged 15–49 years
  Permanent residents of Debre Berhan town for at least six months before data collection
  Willing to participate in the study

The following criteria were set for ruling out the study participants:

  Lactating women who were severely ill and unable to communicate with the data collectors
  Not willing to participate in the study
  Those who could not be found at home after three visits were excluded from the study

### 2.2. Sample Size Determination

The base sample size was calculated for each objective by using the StatCalc application of Epi Info ^TM 7.0.8.3^ (2011) as follows:

   *For the First Objective*. The determination of base sample size was done by using Epi Info based on the assumption that considered an anticipated population proportion of 50% for drivers of food choice with a 95% confidence level and a 5 % margin of error.
 
*For the Second Objective*. From a study conducted in the Republic of Ireland, sex and age had shown a sociodemographic association with healthy value consideration during food choice. Accordingly, women were more concerned about the health value in choosing foods than men with an AOR of 2.68 and 37.6% of men (unexposed subjects) had a healthy eating motivation. Besides, compared to 18–35 years' aged participants, 51–64 and > 64 years' aged participants were more concerned with the health value of foods during food choice with AOR of 2.7 and 6.03. Furthermore, 23.7% of 51–64 and 17.7% of >64 aged groups had the healthy eating motivation (unexposed subjects) [31]. Using Epi Info and taking 95% confidence level, 80% power, 1:1 ratio of the unexposed group and exposed group, outcomes in unexposed groups, and given odds ratios, base sample sizes for those assumptions were calculated as follows. From the calculated base sample sizes, 384 was the largest and used to calculate the final sample size by adjusting the nonresponse rate of 10%.
  Final sample size = [largest base sample size + (largest base sample size^*∗*^ nonresponse rate)]
 
*n* = [(384 + (384*∗*0.10))] = 423

### 2.3. Sampling Procedures

**Figure 1:**
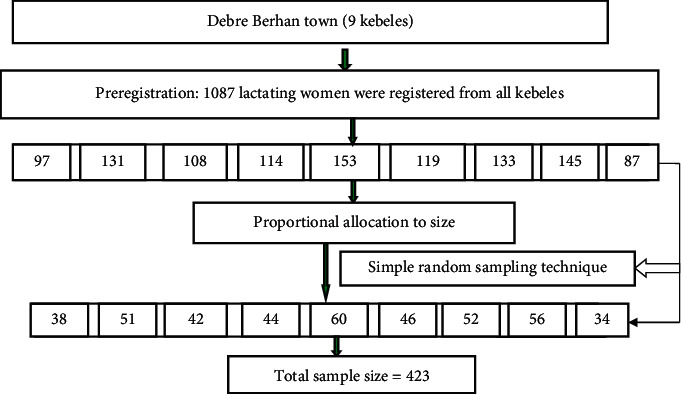
Schematic representation of sampling techniques for studied lactating women (*n* = 423) in the case of central Amhara, Ethiopia, from March to June 2016.

The detailed schematic representation of sampling techniques of the study is presented in Figure 1 below. Debre Berhan town is purposefully selected as a study area and has 9 kebeles. To get representative study samples, all kebeles were used to select study participants. Before the actual data collection, preregistration of lactating women in each kebele was done to count the numbers of lactating women. In all kebeles, the households with lactating women were identified through the house-to-house visit by ketene leaders. In this exercise, a total of 1087 lactating women were registered from the nine kebeles of Debre Berhan town. After house-to-house visit, a sampling frame or a complete list of lactating women was prepared for each kebele. The lottery method was applied to the selected lactating woman from a household that contains more than one lactating woman. Then, the total sample size (423) was proportionally allocated to the number of each kebele's lactating women who were found during preregistration.

To select those proportionally allocated lactating women from each kebele, the simple random sampling technique was applied. Random selections of proportionally allocated lactating women were done by using the planning application of ENA for the SMART computer software program (2007). In doing this, first, a random number table was generated for each kebele based on the given range and required a random number (number of proportionally allocated lactating women) from the range. Using these generated specific random numbers, the corresponding lactating women were marked from the complete list as study participants in each kebele of Debre Berhan town.

### 2.4. Data Collection Method and Instrument

Most important data were collected using an interviewer-administered questionnaire using three trained female data collectors and two supervisors. The interview was conducted at home and it took, on average, 20–25 minutes. The questionnaire is adopted by the researcher from relevant kinds of literature considering the cultural, political, historical, and economic values of the study area.

The questionnaire was separated into four parts: socioeconomic, dietary patterns, food choice behaviors, and physical examination. Physical examination was applied to do the anthropometric measurements such as weight, height, mid-upper arm circumference (MUAC), waist circumference, waist-to-hip ratio, wrist and thigh circumference at the right side, and hip circumference, which were taken from women. Triplicate measurements were done on the same day for each study subject using calibrated equipment and standardized techniques, and the mean of the measures was reported as the final measure.

Furthermore, removing shoes and heavy clothes were done for weight and height measurements. Accordingly, height was measured to the nearest b0.1 cm with barefoot, and weight was measured to the nearest 0.1 kg with light clothing. Body mass index was obtained by dividing the weight in kilogram by the height in meter squared (kg/m^2^). The mid-upper arm circumference was measured by using a nonstretchable MUAC measuring tape. Then, classification was applied using the cut-off point MUAC <21 cm and MUAC ≥21 cm [32].

### 2.5. Data Quality Assurance

To address major areas of bias that can be introduced during the data collection process, the following actions were considered critically. First, the English version of the questionnaire was translated into a survey language which was Amharic and retranslated back to English for consistency by comparing with the original version. Reviewing of the questionnaire was done by senior researchers, and comments were incorporated. Then, the final version questionnaire was prepared and pretested in 22 lactating women to check its understandability by the study participants, the response rate to each question, determine the time required per questionnaire, and the necessary corrections were taken. Three days of training on the objective of the study were prepared by the principal investigator and given to data collectors on interviewing techniques and data collection tools. Site supervision, daily monitoring, and cross-checking of data for completeness and consistency of collected data were carried out by supervisors as well as the principal investigator.

Data on FVs consumption were obtained separately with 24-hour recalling method using questions adapted from the WHO [33]. One standard serving size equals 80 grams and is measured by showing pictorial show cards of common FVs found in the study setting. The picture cards were important to recalling FVs intake and minimizing error in the estimation of standard portions consumed. Medium-size fruits such as orange, apple, banana, and other fruits like half of the avocado, large mango, half cup of fruit juice, one cup of raw green vegetables, half cup for cooking (chopped) vegetables/fruits counted as one portion size. Then, participants who had two daily serving sizes for fruits and three daily serving sizes for vegetables were merged and given a 1 value to denote the FVs consumption category which is important to determine whether each participant met the criterion [20]. The final dichotomous variables were created by classifying participants as “meets criterion-good consumption” and “does not criterion.”

### 2.6. Data Analysis

Data was captured by the researcher using SPSS version 20 computer software program. Descriptive statistics were computed for all relevant variables (socioeconomic characteristics, FVs intake, food choice behavior, and anthropometric indices). Multicollinearity and model fitness were also checked. The nutritional status of women as a dependent variable was classified into BMI <18.5 and BMI ≥18.5 kg/m^2^. To determine the association between predicting food choice behaviors of women, logistic regression was used with both bivariate and multivariate analyses. Candidate variables were selected and transferred to a multivariate analysis by using a preset *P*-value of <0.25. Adjusted odds ratios with 95% confidence interval were the outcomes of logistic regression and reported to quantify the strength of associations. A *P*-value of less than 0.05 was taken as statistically significant motivational behaviors of the outcome variable.

### 2.7. Ethics and Consent

The objective of the study was clarified to Debre Berhan town administrative health care officials for their permission and support. The purpose of the study was explained to the study participants and written consent was taken. The responses were kept confidential by coding. Finally, nutrition information about FVs consumption and its importance was given to women in the study area.

## 3. Result

### 3.1. Sociodemographic Status of Women

A total of 423 lactating women (100% participation) were interviewed in this study. Accordingly, about 63.8% of the respondents had 26–35 years old, 46.1% of respondents had completed higher education (college and above), 81.8% were married, and 53.9% had worked for money/payment (see Table 1). This study also examined the estimated household income earned per month from income-generating activities. The classification was done using the median of monthly income and 50.2% of the respondents earned ≤3500 Ethiopian birr per month.

### 3.2. Anthropometrics Characteristics

About 26.5%, 5%, and 13.5% of women had body weight ≤45 kg, height ≤145 cm, and MUAC <21 cm. About 92.7%, 83.5%, and 75.7% of women had a waist circumference ≤ of 80 cm, waist-to-hip ratio ≤ of 0.8, and the waist-to-height ratio ≤ of 0.5. The prevalence of chronic energy deficiency was 21.7% with a mean value of 21.52 ± 2.71 (see Table 2).

### 3.3. Food Choice Behaviors

The current study provides a basic concept regarding the food choice behaviors of women. This study showed that food selection based on religion was mentioned by over 388 (91.7%) of respondents. Accordingly, lactating women reported price 355 (83.9%), preparation convenience 352 (83.2%), best friend encouragement 344 (81.3%), husband encouragement 300 (70.9%), meal healthiness 280 (66.2%), and daily availability of fruit and vegetable 266 (62.9%) as mainly key drivers (see Table 3).

The perceived price of selected FVs items for those prices concerned with studied lactating women is presented in Table 4. Choosing foods based on taste, its ingredient content, for weight control, medical reason, and based ethical value was mentioned by 237 (56%), 209 (49.1%), 194 (45.9%), 193 (45.6%), and 164 (38.8%) of study respondents, respectively. Among participants who were conscious of weight, food items low in carbohydrate and fat contents were preferred. The ethical concern during food choice signals a common area in origin, environmental acceptance, and official recognition of foods. Avoiding because of nutrient content was mentioned by 144 (34%) of study participants. Choosing foods for mood purposes was the least underlying motives, which accounted for 92 (22%). Choosing foods for feeling good (23.9%) and coping with life (21.7%) were the most important mood options next to keeping alert which accounted for 35 (38%) (see Table 3).

### 3.4. Fruits and Vegetable Consumption

Good consumption of FVs was observed among 37.4% of the studied lactating women (see Table 5), i.e., the prevalence of meeting daily recommendations for daily eating of FVs. Fruits tended to be consumed less frequently than vegetables.

### 3.5. Association between Food Choice Behaviors and FVs Consumption

The primary objective of this study is to investigate the association between food choice behaviors and FVs intake. As a result, to investigate food choice behaviors influencing the FVs consumption of the women, logistic regression analysis was used using both bivariate and multivariate analyses. As a result, healthy meal, ingredient content, avoiding nutrient content, mood, religious influence, husband encouragement, and availability of (FVs and milk and milk products) at the home were significantly associated with the FVs consumption of lactating women during bivariate analysis (*P* < 0.05) (see Table 6).

After adjusting for all candidate variables (preference for a healthy diet, to avoid some nutrient, mood, nutrient content, convenience, price, medical reason, weight control, religion taboo, peer pressure, the influence of the husband, availability of FVs at home, and availability of dairy products at home) in the multivariate logistic analysis; natural content, mood concern, religious influence, husband encouragement, and availability of FVs and milk and milk products were associated with FVs consumption (*P* < 0.05) (see Table 6). Accordingly, among lactating women who were highly motivated to choose a meal based on natural content, mood concern, religious influence, and encouragement by their husband, FVs consumption was significantly higher with AOR (95%) 2.37 (1.39–4.06), 2.29 (1.15–4.56), 2.45 (1.37–4.4), 2.26 (1.04–4.89), and 1.66 (1.16–3.48), respectively.

### 3.6. Association of Food Choice Behaviors and Nutritional Status among Women

Healthy meal motivation, mood, and price concern were significantly associated with the nutritional status of women during bivariate analysis (*P* < 0.05) (see Table 7). After adjusting for all candidate variables in multivariate analysis, healthy meals, price, and mood concern were associated significantly with the nutritional status of women (*P* < 0.05) (see Table 7). Accordingly, for lactating women who were motivated to choose healthy meals and price concerns, normal weight was significantly higher with an AOR of 2.1 (95% CI: 1.21–3.62) and 3.01 (95% CI: 1.32–6.9), respectively. Compared to lactating women, who were concerned to choose food for mood purposes, normal weight was significantly lower among women who were not concerned with an AOR of 0.5 (95% CI: 0.30–0.95).

## 4. Discussion

The present study discusses the nutritional status and FVs intake behaviors of lactating women. Furthermore, as a principal finding, the study also elaborates the influencing contribution of food choice behaviors on nutritional status and FVs intake of women during lactation from central Ethiopia. Accordingly, the FVs consumption trend and nutritional status were determined. As a result, about 37.4% of lactating women had good trends in consuming FVs. However, this finding was higher compared to other studies done in Ethiopia [34], Uganda [35], and Atlanta [36] with a prevalence of 20, 12.7%, and 27.7%, respectively. The prevalence of body mass index less than 18.5 kg/m^2^ among women was 21.7%, which was lower than the finding of Tigray region of Ethiopia (25%) [7], and the Ethiopian Health and Nutrition Research Institute nutrition baseline survey report (28.8%) [37].

Furthermore, the prevalence was much lower than the result of other studies [6, 38, 39], which was about 37.5%. Perhaps, the community tested might be accessible to various health promotions and the kind of nutrition that they would require to maintain their health due to the given time interval. It is also analogous to a study done by Haregu and his colleagues [40].This present study showed that vegetable consumption was higher compared to fruit consumption, which is similar to a study done in SNNPR by Handiso , but the prevalence is still lower [41]. This might be because women from the southern region have access to those agricultural products. However, the prevalence of this finding is not in agreement with a study done by Chen and Gazmararian (2014), which showed low consumption of green salad, carrot, and other vegetables than fruit juice and fruit intake [40].

The main finding of this study was that Debre Berhan women's FVs consumption and nutritional status were associated with food choice behaviors. Among lactating women who were highly motivated to choose a meal for natural content, meals made from natural ingredients were more preferable and perceived to be healthy, including porridge, brown bread, fruits, and vegetables [42]. The likelihood of being in the normal weight among lactating women was directly associated with strong healthy eating motivation. The strong association between healthy meals eating motivation and nutritional status might be explained by the fact that those who had higher motivation had increased and diversified food consumption as compared to those who had no motivation. Since people's motivations towards eating a healthy diet are generally positive to obtain better quality diets which can affect their nutritional status. The finding was inconsistent with Naughton et al.'s study [31].

FVs consumption was significantly higher among women who were concerned about their mood. It contradicted the result explained as emotional eaters consume sweet, fatty foods to regulate their emotions while it was unrelated to the consumption of FVs [43–45]. This could be due to the variation among current and past studied target population. Women encouraged by their husbands had higher FVs consumption. The possible explanation could be social support from within the household and from coworkers was positively associated with improvements in FVs consumption. Because family and friends can be a source of encouragement in making and sustaining dietary change, adopting dietary strategies that are acceptable to them may benefit the individual whilst also having an effect on the eating habits of others [28]. The prevalence of underweight was significantly higher among women who were concerned about stress during food choice. This could suggest that those study participants are affected by their emotions to choose different food items. Psychological stress is a common feature of life and can modify eating behaviors leading to poor quality of food consumption due to mental stress initiated by working under time pressure during peak periods. This increases the risk of poor nutritional status and ultimately jeopardizing their health prognosis and related outcomes [27]. Research has shown that along with easier access and availability of unhealthy foods, bodyweight statuses are closely associated with tension and anxiety within the workplace [46].

Women who were highly concerned with their religion had good FVs consumption since FVs are fasting food groups and preferable for fasting time especially for Orthodox religion which is practiced among studied women. Additionally, FVs consumption was significantly higher among lactating women with home availability of FVs. The availability of healthy food at home and away from home in the community increases the consumption of such foods [47]. FVs consumption was significantly higher among lactating women with home availability of milk and milk products. This could be due to the complementary behavior of women concerning the selection decision of such healthy food groups.

Furthermore, price-concerned lactating women were more likely to be normal in their body weight. It is comparable to the study of Epstein et al., which described the relation of cross-price elasticity with bodyweight [48]. This result suggests that leaner mothers may be more likely to shift purchasing from low energy-dense to high energy-dense foods based on price changes of low energy-dense foods. Other expected reasons could be the purchasing power of women may be limited to the specific food items and influenced by the purchasing frequencies of food items. Additionally, the knowledge of study respondents may affect price over income, i.e., using available resources wisely to choose a balanced diet may be predominated in those lactating women. The less expected association between meal healthy value consciousness and FVs consumption was insignificant and inconsistent with previous studies showing a significant association involving higher FVs consumption and healthy eating motivation [31, 49, 50].

*Strengths and Weaknesses*. The community-based approach, random selection of the study population, and the study samples were selected directly from the targeted population and an interviewer was present, and respondents answering questions about their chosen task were the major strengths of the current study. This may allow generalization since an attempt was made to identify randomized lactating women from the target communities. Since the study consisted of only lactating women, the results may not apply to the general population who were not lactating women. Even if the sample size was considerable, due to resource scarcity, the study respondents were limited to 423 participants. Still, miscounting of eligible women and other biases like social desirability, selection, interviewer, measurement, and information bias could be expected. Particularly, the introduction of partiality is expected during the anthropometric measurement and FVs intake assessment due to measurement and interviewer bias, respectively. Recalling bias, overestimation, and underestimation of food items could be expected at the data collection process. Literature review and discussion were done with other target groups from other countries that might have different settings and this could make a great variation. A limitation of a survey questionnaire is that one can only include and compare a limited number of attributes. Furthermore, cross-sectional data is a limit within itself to determine cause or effect. It relatively indicates a snapshot of a moment in time of the person's daily life.

## 5. Conclusions and Recommendation

FVs consumption and normal weight of women were assessed and it is low in this study. Furthermore, food choice behaviors associated with the consumption of FVs and nutritional status were identified. Accordingly, ingredient content, mood concern, religious concern, and home availability (of milk and milk products and FVs) were associated significantly with FVs intake. Additionally, healthy meal motivation, price, and mood concern were the most important food choice behaviors associated with the nutritional status of lactating women. This research is useful to create insight about food choice behaviors for a group of women with their motivations and lead practitioners to be able to make a recommendation as to what health promotion strategies one should be based on.

## Figures and Tables

**Table 1:** Sociodemographic characteristics of lactating women (*n* = 423) in central Amhara from March to June 2016.

Variables	Frequency (*n*)	Percentage (%)
Age groups	28.79 ± (4.97)^1^	
15–25 years	109	25.8
26–35 years	270	63.8
36–49 years	44	10.4

Educational status		
No formal education	30	7.1
Primary	97	22.9
Secondary	101	23.9
College and above	195	46.1

Marital status		
Married	346	81.8
Other	77	18.2

Head of a family		
Woman	71	16.78
Other	352	83.2

Occupation		
Not having work	195	46.1
Having work	228	53.9

Family size	4.39 ± 1.41	
2–4 persons	240	56.7
≥5 persons	183	43.3

Length of the lactation period		
≤12 months	233	55.08
>13 months	190	44.92

Monthly income in ETB		
≤3500	215	50.2
>3500	208	49.8

*Note*. 1 = mean ± standard deviation. ETB = Ethiopian Birr.

**Table 2:** Anthropometric characteristics of studied women in central Amhara from March to June 2016 (*n* = 423).

Variables	Frequency (*n*)	Percentage (%)
Weight in kg		
≤45	112	26.5
>45	311	73.5

Height in cm		
≤145	21	5
>145	402	95

MUAC in cm		
<21	57	13.5
≥21	366	86.5

Waist circumference in cm		
≤80	392	92.7
>80	31	7.3

Waist-to-hip ratio		
≤0.8	353	83.5
>0.8	70	16.5

Waist-to-height ratio		
≤0.5	320	75.7
>0.5	103	24.3

BMI in kg/m^2^		
<18.49	92	21.7
18.5–24.99	294	69.5
≥25	37	8.8

*Note*. MUAC = mid-upper arm circumference, BMI = body mass index, cm = centimeter, and kg = kilogram.

**Table 3:** Food choice behaviors among lactating women in central Amhara from March to June 2016 (*n* = 423).

Variables	*n*	%	95% CI
Healthy value of a diet			
Yes	280	66.2	61.5–71.7
Vitamin/mineral	128	45.71	
Keeping me healthy	77	27.5	
Reduce adverse risk	15	5.36	
High in protein	43	15.36	
Good for skin, teeth, hair, and nail	2	0.71	
Recommended by physicians	15	5.36	
No	143	33.8	28.3–38.5
Avoiding nutrient content			
Yes	144	34	29.5–39.2
High in fat	75	52.08	
High in CHO	49	34.03	
High in CHO and fat	4	2.78	
High in salt	7	4.86	
Pungent pepper-containing foods	9	6.25	
No	279	66	60.8–70.4
Ingredient content			
Yes	209	49.1	44.8–54.1
Natural ingredients	134	64.1	
Artificial ingredients	75	35.9	
No	214	50.6	45.1–55.2
Price concern			
Yes	355	83.9	80–87.5
No	68	16.1	12.5-6-20
Taste			
Yes	237	56	51.3–60.6
No	186	44	39–48.7
Mood			
Yes	92	22	18–25.3
Helps me cope with stress	20	21.74	
Helps me cope with life	2	2.17	
Helps me relax	11	11.96	
Keeping me alert	35	38.04	
Cheering me up	2	2.17	
Helps me feel good	22	23.91	
No	331	78	74.7–82
Preparation convenience			
Yes	352	83.2	79.7–86.5
No	71	16.8	13.5–20.3
Religion taboo			
Yes	388	91.7	89.1–94.3
No	35	54.4	5.7–10.9
Weight control			
Yes	194	45.9	41.6–50.4
Low in carbohydrate	71	36.6	
Help me to control weight	47	24.23	
Helps me to admire people	3	1.55	
Helps me have a smart weight	6	3.09	
Low in fat	67	34.54	
No	229	54.1	49.6–59.4
Health concern (medical reason)			
Yes	193	45.6	41.1–50.4
No	230	54.4	49.6–58.9
Worry about their health			
Gastric disease	112		
Heart case	21		
Diabetes	24		
Kidney	6		
Allergy	7		
Blood pressure	11		
Cancer	12		
Peer pressure			
Yes	344	81.3	77.3–85.6
No	79	18.7	14.4–22.7
Influence of husband			
Yes	300	86.7	82.8–89.9
No	46	13.3	10.1–17.2
Availability of FVs at working place			
Yes	95	33.8	28.3–39.5
No	186	66.2	60.5–71.7
Availability of FVs at home			
Yes	266	62.9	57.4–67.1
No	157	37.1	32.9–42.6
Availability of soft drink at home			
Yes	147	34.8	30.3–39.7
No	276	65.2	60.3–69.7
Availability of dairy products at home			
Yes	317	74.9	70.7–79
No	106	25.1	21–29.3

**Table 4:** Some of the selected fruits, vegetables, and their perceived price among lactating women who were price-conscious in central Amhara from March to June 2016 (*n* = 355).

Items (kg)	Perceived price											
	Expensive				Cheap				Accessible		Not bought	
	*n*	%	ETB	Mean ± SD	*n*	%	ETB	Mean ± SD	*N*	%	*n*	%
Onion	222	62.54	6–10	11.84 ± 1.67	133	37.46	8–12	10.10 ± 0.89	—	—	—	—
Papaya	236	66.48	13–24	15.21 ± 1.77	8	2.25	14–15	14.50 ± 0.54	—	—	111	31.28
Carrot	136	38.31	4–12	7.82 ± 2.00	200	56.34	3–10	5.77 ± 2.06	19	5.35	—	—
Orange	240	67.6	30–42	35.8 ± 1.77	97	31.6	25–40	33.1 ± 2.64			15	4.22
Garlic	351	98.87	40–70	49.33 ± 5.40	2	0.56	45–48	46.5 ± 2.12	—	—	2	0.56
Banana	219	61.69	10–17	14 ± 1.33	113	31.83	10–16	11.77 ± 1.49	—	—	23	6.48
Tomato	236	66.48	10–18	14.17 ± 0.99	115	32.39	5–16	9.76 ± 2.47	—	—	4	1.13
Mango	250	70.4	30–40	38 ± 1.99	87	24.5	25–40	32.3 ± 2.14			15	4.22
Avocado	272	76.62	12–18	13.93 ± 1.09	5	1.41	13–14	13.8 ± 0.45	—	—	78	21.97
Items in 1 piece									—	—	—	—
Cabbage	126	35.49	5–10	8.91 ± 1.43	213	60	4–10	5.78 ± 1.65	16	4.51	—	—
Items in one handgrip of palm												
Kale	97	27.32	4–10	6.81 ± 2.30	225	63.38	3–10	4.62 ± 1.13	33	9.3	—	—
Lettuce	98	27.61	4–10	7.76 ± 2.35	226	63.66	3–10	4.83 ± 1.09	17	4.79	14	3.94

*Note*. kg = kilogram, SD = standard deviation, ETB = Ethiopian Birr, and –  = no response.

**Table 5:** Prevalence of FVs intake among lactating women in central Amhara from March to June 2016 (*n* = 423).

Daily intake trend in average	Response			
	Yes		No	
	*n*	%	*n*	%
Vegetables (3 servings)	218	51.54	205	48.46
Fruits (2 servings)	161	38.06	262	61.94
Fruits and vegetables (5 servings)	158	37.4	265	62.65

**Table 6:** The association with FVs intake among lactating women in central Amhara from March to June 2016 (*n* = 423).

Food choice behaviors	The choice for FVs intake	
	COR (CI)	AOR (CI)
Preference for a healthy diet	1.91 (1.27–2.89)^*∗∗*^	1.58 (0.91–2.77)^*∗*^
To avoid some nutrient	1.73 (1.12–2.66)^*∗∗*^	1.68 (0.97–2.91)^*∗*^
Mood	2.07 (1.23–3.48)^*∗∗*^	2.29 (1.15–4.56)^*∗∗*^
Taste	1.11 (0.75–1.65)	
Nutrient content	3.19 (2.11–4.83)^*∗∗*^	2.37 (1.39–4.06)^*∗∗*^
Convenience	1.58 (0.94–2.64)^*∗*^	1.06 (0.51–2.19)
Price	0.6 (0.34–1.07)^*∗*^	0.61 (0.29–1.26)^*∗*^
Medical reason	1.05 (0.7–1.56)^*∗*^	0.85 (0.51–1.4)
Weight control	1.3 (0.88–1.94)^*∗*^	0.96 (0.58–1.59)
Ethics	1.01 (0.68–1.52)	
Religion taboo	1.81 (1.18–2.79)^*∗∗*^	2.45 (1.37–4.4)^*∗∗*^
Peer pressure	1.62 (0.99–2.66)^*∗*^	1.12 (0.58–2.14)^*∗*^
Influence of husband	2.81 (1.49–5.28)^*∗∗*^	2.26 (1.04–4.89)^*∗∗*^
Availability of FVs at working place	1.3 (0.75–2.26)	
Availability of FVs at home	2.3 (1.53–3.45)^*∗∗*^	1.66 (1.16–3.48)^*∗∗*^
Availability of dairy products at home	2.13 (1.36–3.33)^*∗∗*^	1.6 (1.39–2.74)^*∗∗*^

*Note*. FVs = fruits and vegetables, ^*∗∗*^ = *P* < 0.05, ^*∗*^*P* ≤ 0.25, COR = crude odds ratios, AOR = adjusted odds ratio, and CI = confidence interval.

**Table 7:** The food choice behaviors associated with the nutritional status among lactating women in central Amhara from March to June 2016 (*n* = 423).

Variables	Nutritional status			
	Undernourished (BMI *<*18.5* *kg/m^2^)	Normal (BMI ≥18.5 kg/m^2^)	COR (CI)	AOR (CI)
Avoiding food as not good for a child	59	233	0.96 (0.58–1.61)	
	35	96		
Healthy meal	71	209	1.97 (1.156–3.37)^*∗∗*^	2.1 (1.21–3.62)^*∗∗*^
	21	122		
Avoid nutrient	30	114	0.92 (0.56–1.51	
	62	217		
Mood	13	79	0.53 (0.28–0.99)^*∗∗*^	0.5 (0.30–0.95)^*∗∗*^
	79	252		
Taste	50	187	0.92 (0.58–1.46)	
	42	144		
Natural content	164	167	0.79 (0.5–1.26)	
	51	41		
Preparation convenience	73	279	0.72 (0.4–1.29)	
	19	52		
Price	85	270	2.74 (1.21–6.22)^*∗∗*^	3.01 (1.32–6.9)^*∗∗*^
	7	61		
Medical reason	36	157	0.71 (0.45–1.14)^*∗*^	0.65 (0.40–1.06)
	56	174		
Weight	40	154	0.88 (0.56–1.41)	
	52	177		
Religion influence	87	301	1.73 (0.65–4.6)	
	5	30		
Friend encouragement	72	272	0.78 (0.44–1.38)	
	20	59		
Husband encouragement	44	256	0.49 (0.23–1.02)^*∗*^	0.81 (0.33–2.86)
	12	34		
Availability of soft drink at home	32	115	0.46 (0.25–0.86)^*∗∗*^	0.47 (0.2–1.14)^*∗*^
	60	215		
Availability of FVs at home	54	212	0.8 (0.5–1.28)	
	38	119		
Availability of milk and milk products at home	64	253	0.71 (0.42–1.18)^*∗*^	2.4 (0.67–8.66)^*∗*^
	28	78		
Advertisement	30	104	1.1 (0.65–1.73)	
	62	227		
Nutrition books, journals, and magazines	22	73	1.11 (0.64–1.92)	
	70	258		

*Note*. FVs = fruits and vegetables, ^*∗∗*^*P* ≤ 0.05, ^*∗*^*P* ≤ 0.25, AOR = adjusted odd ratio, BMI = body mass index, CI = confidence interval, and COR = crude odds ratio.

## Data Availability

The datasets used and analyzed to support the findings of this study are available from the corresponding author upon reasonable request.

## Abbreviations

AOR: Adjusted odds ratio
BMI: Body mass index
CI: Confidence interval
Cm: Centimeter
COR: Crude odds ratio
CSA: Central Statistics Agency
EDHS: Ethiopia Demographic and Health Survey
EMDHS: Ethiopia Mini Demographic and Health Survey
ETB: Ethiopian birr
FAO: Food and Agriculture Organization
FMOH: Federal Minister of Health
FVs: Fruits and vegetables
Kg: Kilogram
NCDs: Noncommunicable diseases
SPSS: Statistical Package for Social Sciences
SD: Standard deviation
UNICEF: United Nations International Children's Emergency Fund
USAID: United States Agency for International Development
WHO: World Health Organization.
